# Adverse drug events—Analysis of a decade. A Portuguese case-study, from 2004 to 2013 using hospital database

**DOI:** 10.1371/journal.pone.0178626

**Published:** 2017-06-02

**Authors:** Gianina Scripcaru, Ceu Mateus, Carla Nunes

**Affiliations:** 1 Escola Nacional de Saúde Pública, Universidade NOVA de Lisboa; Lisbon, Portugal; 2 Amgen Biofarmaceutica, Lda.,Lisbon, Portugal; 3 Health Economics Group Division of Health Research, Lancaster University, Lancaster, United Kingdom; 4 Centro de Investigação em Saúde Pública, Universidade NOVA de Lisboa; Lisbon, Portugal; University of Brescia, ITALY

## Abstract

**Purpose:**

The goal of this study was to characterise adverse drug events (ADE), including both adverse drug reaction (ADR) and accidental poisoning by drugs (AP), considering age, gender, length of stay (LOS), number of deaths and year, during the period 2004–2013. Additionally distributions of the ten’s most frequent ADR and AP were characterized, considering age-group and gender.

**Methods:**

A retrospective descriptive nationwide study was conducted, based on the hospital discharges database in Portugal from 2004 to 2013, using ICD-9. Events were identified based on the following codes: from E930 to E949.9 and from E850 to E858.9.

**Results:**

A total of 9 320 076 patients were discharged within this period, with 133 688 patients (1.46%) having at least one ADE, 4% of them related with AP. The mean age of these patients was 63.79 years (SD 21.31), 54.50% were female and the mean LOS was 14.05 days (SD 22.19). Patient with AP had a mean age of 41.06 years (SD 34.05), 54.70% were female and LOS was 7.15 days (SD 19.42). We have identified 10.691 deaths that represent 8.00% from the total of patients with an ADE. The patients above 65 years were more affected by ADR and children below 18 were more affected by AP.

**Conclusion:**

In the last decade an increasing trend of ADR were observed and an AP pattern relatively stable. Elderly people and children were the age groups most affected. Antibiotics (in ADR) and benzodiazepine-based tranquilizers (in AP) were the major problems. This is a huge, increasing and challenging problem. Further research, using individual and contextual risk factors should be developed to understand spatiotemporal variability, promoting tailored interventions, within and across countries.

## Introduction

Patient safety and the safe use of medicines are priority issues in modern medicine. Pharmacovigilance activity allows continuous monitoring of medicines by identifying serious consequences and accidental exposure to the drugs as well as identifying the drugs that cause adverse drug reactions and their supervision. Current efforts worldwide are now being developed to reduce morbidity and mortality related to drugs leading to widening the goal of pharmacovigilance including quality analysis, prescribing errors, dispensing and administration of the drugs [1].

The definition of adverse events related with the drugs (ADE) is not a fixed and stable concept in time, reflecting the complexity and manifold settings considered in patient safety and pharmacovigilance [2], and has been recently changed [3]. Therefore, lately and using a more comprehensive approach, the studies of the ADE tend to include both poisoning by drugs (AP) and adverse reaction of drugs (ADR) [4, 5].

Determining the incidence of adverse drug reactions may be difficult, because frequently drugs are not recognized as the cause of symptoms or diseases [6]. Previous studies have shown the rate of ADR in hospital inpatients ranging from 0.8% to 26.1% [7–11]. Also it was found that 1.8% to 12.8% of ADR can lead to hospitalization [12–15]. McDonnell *et al*. in their study showed that 67% of hospitalizations caused by adverse drug reactions were due to inadequate monitoring of the patient [16]. Mortality due to adverse drug reactions is a concern, the mean death rate for this cause can reach 5% for all in hospital in-patients with a drug related problem [17]. Many of the adverse reactions are associated with specific factors related with the patient and/or with the drug. Other factors that contribute to adverse reactions are related with the accuracy of prescribing, dispensing and administration the drugs [18].The risk factors related with patient include older age, female gender, drugs in therapy and the number of associated comorbidities [19–21].

In a study in Portugal from 2013, Miguel *et al*. stated that ADR rate is 1.26% from all discharges and 97.3% of those occurred during hospitalization, considering the period 2000–2009 [22]. These earlier findings leads us to others imperatives, more comprehensive and updated questions in order to promote the development of health policies with optimal effects on the patient: What kind of ADE is more frequent in Portugal and who is more affected? Is this an increasing problem?

The main goal of this study was to analyse ten-year’s time period in Portugal hospitalizations, characterizing the adverse events related with drugs. Global analyses, by type of ADE (ADR and AP), were done considering gender and age: Additionally, specific analyses were focused on ten´s most frequent ADR and AP.

## Methods

We included in this analyse ADE defined as injuries resulting from the use of prescription and over-the-counter medications for medical intervention, which includes adverse drug reactions and medication error and excludes administration of the wrong medication, intentional overdoses or use of illicit substances, in agreement with the literature [4, 5, 23–25].

Using the Portuguese impatient database was performed a retrospective nationwide study from 2004 to 2013 analysing patients with ADE by age, gender and the distribution of the ten most frequent ADR and AP by age group and gender. The database aggregates data from each public hospital and was obtained from Administração Central do Sistema de Saúde (ACSS), the Ministry of Health’s Central Authority for Health Services. The information is organize in Diagnosis Related Groups (DRG), a classification system of hospitalized patients which groups inpatients clinically coherent and similar from the prospective of consumption of resource, using the International Classification of Diseases– 9th Revision (ICD-9). In Portugal all coders are physicians, who perform a standardized national course and examination. Additionally, this database is frequently evaluated by external auditors, to ensure high quality and to potentially detect and correct errors. This database contains information on anonymized patient identification, episode, and process number and the variables in used were: year, gender, age, discharge LOS, discharge situation, up to 20 diagnoses (principal and 19 secondaries), up to 20 procedure and up to 20 external causes (E codes). Ethics committee approval and informed consent were not required, as data was based on an Official Dataset and was previously anonymized. The unit of analysis is the patient discharged, this means that a patient who is admitted to the hospital multiple times is accounted for each time separately. All outpatient episodes are excluded from the analyses.

Based on literature, following E codes were used from ICD-9 E930 to E949.9 of ADR and E codes from E850 to E858.9 (specifically for AP) [13, 22, 25].

Data were analyzed using SPSS v21 (IBM Corporation) and a statistical significance level of 5%. Statistical analyses, after descriptive analyses, were based in Chi-square or Fisher’s test (for categorical variables) and Student’s t-test (or Mann-Whitney, depending if continuous variables were normally distributed or not). The variable age was grouped into 9 groups, commonly used in the ADE literature.

## Results

There were 9 320 076 patients discharged from hospital between 2004 to 2013, and 133 688 patients present at least one adverse drug event (rate 1.46%), being the majority ADR (128 604, 96%) and accidental poisoning being responsible for 5 084 cases (4%). A total of 1 699 534 (18.24%) patient were admitted to the hospital multiple times, and of this 12 468 (9.33%) are related with ADE. The mean number of different diagnoses codes per inpatient case was 5.1 for ADR and 3.6 for AP.”

*Table 1*presents the characteristics of the population with ADE (also considering subgroups) in comparison with all other discharged. In all discharge most are women, the mean age is 48.28 years and we identify 436 353 deaths which represent 4.70% of all episodes. Comparing the patient with ADE with all inpatients, they are older (mean age 62.93) have higher LOS (mean 13.78), higher death rate (8.0%) and lower percentage of women (54.50).

**Table 1:** Characterization of discharges by ADE, ADR & AP, considering year, age, gender, length of stay and death, 2004–2013.

	All discharges	ADE (E codes)		
		Total	ADR	AP
**Total no.**	9 186 388	133 688	128 604	5 084
**YEARS**				
2004	969 892	11.007 (1.13%)	10 442 (1.08%)	565 (0.06%)
2005	930 271	9 996(1.07%)	9 444 (1.02%)	552 (0.06%)
2006	917 632	11 464(1.25%)	10 911 (1.19%)	553 (0.06%)
2007	906 352	11 719(1.29%)	11 217 (1.24%)	502 (0.06%)
2008	930 538	11 772(1.27%)	11 271 (1.21%)	501 (0.05%)
2009	917 830	12 881(1.40%)	12 404 (1.35%)	477 (0.05%)
2010	933 621	14 325(1.53%)	13 773 (1.48%)	552 (0.06%)
2011	892 948	14 910(1.67%)	14 320 (1.60%)	590 (0.07%)
2012	912 222	16 864(1.85%)	16 560 (1.82%)	304 (0.03%)
2013	875 082	18 750 (2.14%)	18 262 (2.09%)	488 (0.06%)
**Mean age**	48.28 (27.93 SD)	62.93 (22.36 SD)	63.79 (21.31 SD)	41.06 (34.05 SD)
	(CI 48.26–48.30)	(CI 62.81–63.05)	(CI 63.67–63.91)	(CI 40.12–41.99)
**Age group**				
0–18	1 645 254	9 121 (0.55%)	7 101 (0.43%)	2 020 (0.12%)
19–40	1 910 377	12 030 (0.63%)	11 476 (0.60%)	554 (0.03%)
41–65	2 411 018	36 600 (1.52%)	35 933 (1.49%)	667 (0.03%)
>65	3 219 735	75 937 (2.36%)	74 094 (2.30%)	1 843 (0.06%)
**Gender**				
M	4 107 000 (44.70%)	60 783 (1.48%)	58 478 (1.42%)	2 305 (0.06%)
F	5 079 252 (55.30%)	72 905 (1.44%)	70 126 (1.38%)	2 779 (0.05%)
**Mean LOS (days)**	7.04 (24.22 SD)	13.78 (22.13 SD)	14.05 (22.19 SD)	7.15 (19.42 SD)
	(CI 7.03–7.06)	(CI 13.67–13.90)	CI 13.93–14.17)	(CI 6.61–7.68)
**Death**	436 353 (4.70%^1^)	10 691 (8.0%^2^)	10 463 (8.10%^3^)	228 (4.50%^4^)

^1^Percent of all inpatients

^2^Percent of all ADE

^3^Percent of all ADR

^4^Percent of all accidental poisonings; LOS–Length of stay; SD- Standard Deviation; CI– 95% confidence interval. Note: Some missing were observed (136 in gender and 4 in age)

When comparing the discharges with all ADE, ADR and AP all results are statistical significant (p<0.001).

By year the analysis shows a slight increase in the number (and proportion) of all ADE in general, with an increasing pattern in ADR and AP more stable in the period studied. An increase of 70.34% for ADE was observed along the period, with 11 007 events in 2004 and reaching 18 750 in 2013.

By age group, the highest number of ADR was observed in patients with more than 65 years (rate 2.58%) and for AP the most are children below 18 years (rate 0.12%).

In all discharges, the majority (absolute number) were women as well in all in ADE, ADR and AP. All differences presented in this table were statistically significant (p<0.001).

Tables 2 and 3 illustrate the distribution of the ten most frequent drug classes associated with ADR and AP by gender and age class. This ten ADEs represents more than 80% of all ADR and AP in each group. Patients over 65 are the most affected by ADR to anticoagulants and the others age groups are mainly affected by ADR to antineoplastic and immunosuppressive drugs. For AP the children below 18 are the principal affected and the most frequents drugs responsible for AP are benzodiazepine-based tranquilizers (E853.2). Overall, in ADR subgroup the women are predominant, as expected considering the percentage of women in all ADR (55% of women). Although in one case we find a similar distribution between men and women (E933.1). For AP in three cases (E858.1, E850.2, E850.4) we find more men than women.

**Table 2:** Distribution of ten most frequent ADR by gender, 2004–2013.

	E933.1	E934.2	E932.3	E942.1	E932.0	E930.8	E942.0	E944.4	E939.4	E935.6
**AGE CLASS**										
0–18	6592 (16.52%)	44 (0.26%)	72 (0.57%)	12 (0.14%)	219 (2.89%)	324 (4.76%)	20 (0.34%)	6 (0.13%)	73 (2.03%)	59 (2.11%)
19–40	5714 (14.32%)	295 (1.77%)	204 (1.61%)	16 (0.19%)	564 (7.44%)	962 (14.14%)	49 (0.84%)	65 (1.46%)	158 (4.40%)	236 (8.44%)
41–65	17222 (43.16%)	2849 (17.12%)	1830 (14.49%)	371 (4.46%)	2768 (36.51%)	2071 (30.44%)	830 (14.23%)	643 (14.42%)	604 (16.82%)	810 (28.96%)
>65	10373 (26.00%)	13455 (80.84%)	10527 (83.33%)	7921 (95.20%)	4030 (53.16%)	3447 (50.66%)	4933 (84.59%)	3744 (83.98%)	2757 (76.75%)	1692 (60.49%)
**GENDER**										
M	20 304 (50.89%)	7 641 (45.91%)	5 021 (39.68%)	2 289 (27.51%)	3 421 (45.13%)	3 352 (49.27%)	2 625 (44.97%)	1 732 (38.85%)	1 249 (34.77%)	1 323 (47.30%)
F	19 597 (49.11%)	9 002 (54.09%)	7 612 (60.32%)	6 031 (72.49%)	4 160 (54.87%)	3 452 (50.73%)	3 212 (55.03%)	2 726 (61.15%)	2 343 (65.23%)	1 474 (52.70%)
**TOTAL**	39 901 (100%)	16 643 (100%)	12 633 (100%)	8 320 (100%)	7 581 (100%)	6 804 (100%)	5 837 (100%)	4 458 (100%)	3 592 (100%)	2 797 (100%)

Note: E933.1 Antineoplastic and immunosuppressive drugs; E934.2 Anticoagulants; E932.3 Insulins and antidiabetic agents; E942.1 Cardiotonic glycosides; E932.0 Adrenal cortical steroids; E930.8 Other specified antibiotics; E942.0 Cardiac rhythm regulators; E944.4 Diuretics; E939.4 Benzodiazepines; E935.6 Antirheumatics.

**Table 3:** Distribution of ten most frequently AP by gender, 2004–2013.

	E853.2	E858.3	E858.2	E855.0	E858.1	E858.0	E850.2	E854.0	E850.4	E853.8
**AGE CLASS**										
0–18	569 (37.21%)	85 (14.81%)	31 (6.83%)	116 (42.18%)	227 (84.39%)	46 (20.26%)	33 (15.42%)	122 (59.22%)	136 (70.47%)	121 (66.85%)
19–40	77 (5.04%)	1 (0.17%)	10 (2.20%)	33 (12.00%)	7 (2.60%)	14 (6.17%)	88 (41.12%)	12 (5.83%)	30 (14.54%)	4 (2.21%)
41–65	195 (12.75%)	25 (4.36%)	66 (14.54%)	62 (22.55%)	16 (5.95%)	32 (14.10%)	48 (22.43%)	30 (14.56%)	17 (8.81%)	19 (10.50%)
>65	688 (45.00%)	463 (80.66%)	347 (76.43%)	64 (23.27%)	19 (7.06%)	135 (59.47%)	45 (21.03%)	42 (20.39%)	10 (5.18%)	37 (20.44%)
**GENDER**										
M	538 (35.01%)	381 (48.69%)	208 (45.89%)	135 (48.75%)	14232 (53.33%)	109 (48.40%)	135 (61.11%)	92 (43.52%)	82 (59.55%)	80 (43.39%)
	991 (64.99%)	193 (51.31%)	246 (54.11%)	140 (51.25%)	127 (46.67%)	118 (51.60%)	79 (38.89%)	114 (56.48%)	111 (40.25%)	101 (56.61%)
**TOTAL**	1529 (100%)	574 (100%)	454 (100%)	275 (100%)	269 (100%)	227 (100%)	214 (100%)	206 (100%)	193 (100%)	181 (100%)

Note: Acc. poisn. by E853.2 benzodiazepine-based tranquilizers; E858.3 agents primarily affecting cardiovascular system; E858.2 agents primarily affecting blood constituents; E855.0 anticonvulsant and anti-parkinsonism drugs; E858.1 primarily systemic agents; E858.0 hormones and synthetic substitutes; E850.2 other opiates and related narcotics; E854.0 antidepressants; E850.4 aromatic analgesics, not elsewhere classified; E853.8 other specified tranquilizers

For ADR cause by antineoplastic and immunosuppressive drugs (E933.1) and specified antibiotics (E930.8) the distribution is approximately identic between men and women, while for ADR cause by cardiotonic glycosides (E942.1) the difference between genders are important, with more than 70% of women in this category.

We find similar distribution between genders in AP caused by agents primarily affecting the cardiovascular system (E858.3) and anticonvulsant and anti-parkinsonism drugs (E855.0) and hormones and synthetic substitutes (E858.0). While in AP caused by benzodiazepine-based tranquilizers (E853.2) more than 60% are women, in AP caused by opiates and related narcotics (E850.2) and aromatic analgesics (E850.4) more than 60% are men.

## Discussion

The goal of this study was to analyze the ADE in Portugal using the hospital discharges database from 2004 to 2013. As far as we know, this was the first study in Portugal that have identified the most frequent ADEs, including ADR and AP, and considering age, gender, LOS and death. A total of 133 688 ADE was found, being 128 604 ADR (96%). There was an increase in the number of ADE from 11 007 in 2004 to 18 750 in 2013 (global rate 1.46%). In Europe, studies using administrative databases have shown that the rate of adverse drug reactions are between 0.8% - 1.83% [11, 13, 26]. The systematic review of Cano et al about the ADE in hospitals found that the proportion of patients with ADEs ranged from 2.15% to 19.2% in Europe [27]. These results are in line with the systematic review of Miguel et al, with a mean of 16.88% of ADR in hospitalized patients[28]. Studies based on administrative database methodology usually present higher rates of ADEs comparing with spontaneous reporting, however lower when comparing with prospective monitoring and intensive monitoring.

It is well established that pharmacokinetics and pharmacodynamics behavior of drugs is influenced by multiple factors, such as factors related to the patient's pathology and diseases, physiological factors, age, gender, administration of other medicines and lifestyle. [19–21]. Additionally, the variety of these results can be explained by the fact that they have used different definitions and methods for identifying adverse drug events (E code and other validation codes).

Considering age, results have shown that patients over 65 years were more affected by ADE in general. ADR followed the same distribution of ADE, with high number of ADR in older patients. Nevertheless AP were more frequently in children until age 18 (with approximately 40% of all AP).

*Routledge et al*. indicate that babies and elderly patients are prone to develop adverse reactions due to pharmacokinetic processes. First due to incomplete maturation of systems that participate in these processes or an incomplete evaluated capacity to metabolize the drugs, and second, because of changes that occur with age [29, 30].

In children, our study shows that antibiotics are the principal drug class responsible for ADR and benzodiazepine for AP. About the risk of ADR in childrens, a systemic review of Smyth, R. M. *et al*. found that anti-infectives and anti-epileptics are in the most frequently drugs involved in the occurrence of that kind of events[31].

We have identified elderly patients as the most affected group by ADR and the second most affected by AP. Many factors can be associated with ADRs in elderly, although the polypharmacy and comorbidities are the most identified in literature [32–39]. Other factors like, drug interaction [40] pharmacokinetic changes [41], overdoses, use of inappropriate drugs, improper administration, inadequate monitoring in administration period, unmanaged dose, mistaken frequency or known allergy and no preventive therapy were also identified as possible factors [42–45].

With exception to E933.1 –ADR to antineoplastic and immunosuppressive drugs, with the highest rate in age class from 41 to 65, all other ADR are more frequent in patients up to 66 years. Beside E-933.1, we identified the E934.2 –ADR to anticoagulants, E933.1 –ADR to antineoplastic and immunosuppressive drugs, E934.2 –ADR to anticoagulants and E932.3 –ADR to insulins and antidiabetic agents as the principals responsible for ADR in patient up to 66. Our results are consistent with those presented in other studies that have identified antibiotics, cardiovascular drugs, antidiabetic agents and insulin, non-steroidal anti-inflammatory drugs and psychotropic drugs as risk factors for ADR in elderly populations. [12, 33, 37, 46, 47].

In absolute terms we found more women with ADE than men (the women are more hospitalized) but the rate of ADE is higher in men (1.48%) than in women (1.44%). Similar situation is verified for the ADR (rate 1.42% for men *vs* 1.38% for women) and for AP (rate 0.06% for men *vs* 0.05% for women). These results are not in agreement with published results [14, 48], but Miguel *et al*. in their study found a similar result for the period 2000–2009 for Portugal [22].

Based on a systematic review, LOS results have shown an excess in the group of ADE, an expected situation, but with a higher difference between the subgroup of patient with ADR comparing with the LOS of patients hospitalized for other reason [49]. Specifically in case of ADR, our result show an increase of LOS, from 13.70 to 14.05 days, when comparing with the similar study release in Portugal [22]. However, the mortality rate in patient with ADE is higher compared with the patient without ADE. Our results show that 8% of ADE had a fatal outcome that represents 0.11% of all discharges. These results are consistent with those obtained by Pirmohamed *et al*. (0.15%) in England [14] and and Carrasco-Garrido *et al*. (0.1%) in Spain. However this rate is lower than obtained by Lazarou (0.31%) in meta-analyses [50].

We recognize the limitations in our study: first the incomplete information or potential errors in filling out in the hospital database. Second, possible non-identifications of the ADE by the doctors can be problematic. The relatively low reported frequency of ADEs rate can be related also with underreporting [51] or “coding creep”, once in Portugal the coding databases are used for reimbursement [52]. Several studies broadcast the big problems of underreporting of ADEs [51, 53], and educational programs were implemented to intensify ADR reporting all over the Europe [54–58].More detailed studies must be done in order to evaluate this specific issue.

*Dormann et al*. and *Azaz-Livshits et al*. demonstrated that 57% of ADR weren’t recognized before the hospital admission and 47.5% during the discharges [6, 59]. In this analyze we assumed the homogeneity in codification over the years. If this situation is not verified, this bias can affects study results. In a hospital setting and in a nationwide scope, this study have identified the ten most frequent ADE, considering independently ADR and AP, correspondent risk groups (age and gender) and temporal evolution. This new knowledge sustains that this is an increasing problem, and can support the development of new tailored mechanisms (interventions or protocols), for each type of ADE and risk group, in order to minimize these adverse events and consequences (namely death and financial issues associated with hospitalizations).

## Conclusion

The number of ADE is increasing in last decade in Portugal, with the subgroup of AP more stable. The elderly people and the children are the age groups most affected. The most frequently ADR in Portugal are related with antineoplastic and immunosuppressive drugs and the most frequent AP are related with benzodiazepine-based tranquilizers. The impact of ADE is huge and presents an increasing pattern. Future research using other individual (comorbidities, concomitant medication, life style or genetic predisposition) and contextual risk factors (possible regional variability related with health services) can be helpful to explain this spatiotemporal variability in order to promote local tailored and updated actions of prevention.
